# Straw Compost Products Improve Corn Growth in Association with Rhizosphere Microbial Community in Acidic Soil

**DOI:** 10.3390/plants15060879

**Published:** 2026-03-12

**Authors:** Tongyu Feng, Xin Wang, Chao Wang, Renfang Shen

**Affiliations:** 1 State Key Laboratory of Soil and Sustainable Agriculture, Institute of Soil Sciences, Chinese Academy of Sciences, No. 298 Chuangyou Road, Nanjing 211135, China; vonty@issas.ac.cn (T.F.); wangxin@neau.edu.cn (X.W.); rfshen@issas.ac.cn (R.S.); 2 University of Chinese Academy of Sciences, Beijing 100049, China; 3 College of Resources and Environment, Northeast Agricultural University, Harbin 150030, China

**Keywords:** organic amendment, microbial network, nutrient cycling, 16S rRNA sequencing, sustainable agriculture

## Abstract

Straw compost products are considered an excellent organic amendment for acidic soils, yet their effectiveness and microbial associations remain poorly understood. This study employed a pot experiment to evaluate the effects of straw compost products from six crops (corn, soybean, wheat, rice, peanut, and canola) on corn growth and nutrient uptake, soil physicochemical properties, and microbial community in an acidic red soil and examined how microbial community changes relate to plant performance. The results showed that straw compost products significantly enhanced corn growth and contents of nitrogen, phosphorus, and potassium in the aboveground tissues, except for wheat and canola straw. Compost products also improved availability of soil nutrients to varying degrees and affected the bacterial community structures in bulk and rhizosphere soils. There were significant differences in the improvement effects among straw types, with leguminous crops being better than cereal crops. Corn growth was closely correlated with increased soil organic carbon. The influence of the rhizosphere on bacterial communities was stronger than that of straw compost type. The dominant phyla *Actinobacteriota* and *Patescibacteria* were key bacterial groups positively associated with corn nutrient uptake in the rhizosphere. Compared to the bulk network, the rhizosphere microbial co-occurrence network exhibited higher modularity and a greater proportion of positive edges, suggesting a more cooperative interaction pattern. The influence of compost products might be associated with distinct nitrogen and phosphorus transformation pathways. Overall, this study clarifies the differential effects of straw compost products on acidic soil improvement and reveals strong associations between rhizosphere microorganisms and crop nutrient uptake.

## 1. Introduction

As a major agricultural country, China generates large amounts of crop straw, with the total production in 2024 estimated at approximately 885 million tons. This primarily includes corn, rice, wheat, canola, soybean, and peanut [1,2]. Improper disposal of straw not only causes resource waste but also leads to environmental pollution risks. Compared to landfilling and incineration, composting is widely recognized as a cost-effective and environmental strategy for straw management [3]. Returning straw compost products to fields effectively enhances soil organic carbon sequestration [4] and increases soil nutrient content and water-holding capacity [5], as well as boosts soil microbial abundance and functionality [6]. The benefits of straw compost products ultimately improve crop yield and quality and contribute to the sustainability of agricultural ecosystems.

Crop straws exhibit substantial diversity in their characteristics, including nutrient content, structural morphology, and cellulose composition, which in turn determine variations in compost product quality. For example, legume straw generally contains higher nitrogen (N) levels than cereal straw, resulting in correspondingly higher N content in compost products [7]. Among cereal crops, compost products derived from corn often exhibit more N content than wheat and rice [8]. Previous research largely focused on the effects of exogenous additives and composting conditions on straw compost quality, as well as on the application of straw as a co-substrate in manure composting [9]. Owing to the high C/N ratio and contents of cellulose and lignin, straw compost products show the differences in organic matter composition and nutrient availability with manure-based composts. Furthermore, composting alters microbial community structure and species interactions within the materials, thereby influencing the function of microbial communities in the compost products [10] and the returned soil [11]. Current research rarely focuses on crop straw as the primary composting material, and systematic comparisons among straw compost products derived from different crop residues remain limited. In particular, the different effects of compost products on crop growth and nutrient uptake has not been systematically evaluated.

Acidic soils (pH < 5.5) are widely distributed in China, covering approximately 250 million hectares. Acidic soils are typically characterized by low pH, nutrient deficiencies, and low organic matter content, which severely limit improvements in crop yield and quality [3,12]. Over the past three decades, soil acidification has been accelerated due to the extensive input of chemical N fertilizers [13]. Straw compost products, rich in organic matter and nutrients, can partially substitute for chemical N fertilizers to improve acidic soils. The improvement of soil biological functions by straw compost products has received attention. Its application has improved the microbial community structure and enhanced microbial diversity and functional activity in Mollisols [14,15]. However, in acidic soils, the focus has been on the ecological effects of direct straw return, which was been shown to enhance nutrient availability and reshape soil microbial communities [16]. As a vital component of ecosystems, microbial communities are closely associated with N and phosphorus (P) transformation processes in acidic soils, primarily through microbial-mediated mineralization and solubilization pathways [17,18,19]. Therefore, elucidating the associations between microbial communities and nutrient uptake under compost product application is of great significance for enhancing the productivity of acidic soils.

In soil–plant ecosystems, the rhizosphere represents the immediate interface between plant roots and soil, where nutrient availability and microbial communities directly affect plant growth and health [20]. The microbial community composition in the rhizosphere exhibits distinct differentiation from bulk soil, supporting higher functional activity. This is largely influenced by root exudates through the recruitment, selection, and stimulation of specific microorganisms [21]. In agronomic practices such as fertilization or amendment application, bulk soils are influenced solely by the added substances, whereas the rhizosphere environment emerges from the combined effects of plant growth and external substances. Studies demonstrated that rhizosphere effects exert a stronger influence on soil microbes than agronomic practices, yielding insights of microbial mechanisms being distinct from bulk soils [22]. Consequently, rhizosphere microbes should warrant particular attention when agronomic practices enhance crop productivity.

In this study, we hypothesize that straw compost products derived from different crops exhibit varying effects on improving acidic soils, and the differences are closely associated with changes in soil nutrients and rhizosphere microbial communities. Corn (*Zea mays* L.) is a staple crop in the acidic soil regions of southern China [12,23]. Using pot experiments, we selected six major crop types for straw composting and systematically evaluated the improvement effects of straw compost products on acidic soils through corn growth and nutrient uptake and soil nutrients and microbial communities in bulk and rhizosphere soils. This study aims to (1) determine the differences in improvement effects among various straw compost products on acidic soil, (2) examine the differential responses of soil microbial communities to straw compost products, and (3) explore the associations between rhizosphere microbial communities and crop growth under the improvement of straw compost products. This study provides scientific guidance for the application of straw compost products in acidic soils.

## 2. Results

### 2.1. Physicochemical Characteristics of Crop Straws After Composting

The pH of the straw compost products ranged from 7.61 to 10.17 (Table 1), indicating generally alkaline characteristics. SOC contents were relatively consistent among compost types, ranging from 241.20 to 261.14 g kg^−1^. In contrast, nutrient levels varied markedly; rice straw compost exhibited the highest TN, TP, and TK contents. Conversely, wheat and canola composts showed relatively lower TP contents, with wheat compost also displaying the lowest TN. The C/N ratio ranged from 14.13 (rice) to 27.96 (wheat), reflecting significant variation in maturity and substrate composition among the straw types.

### 2.2. Corn Growth and Soil Physicochemical Properties

**Figure 1:**
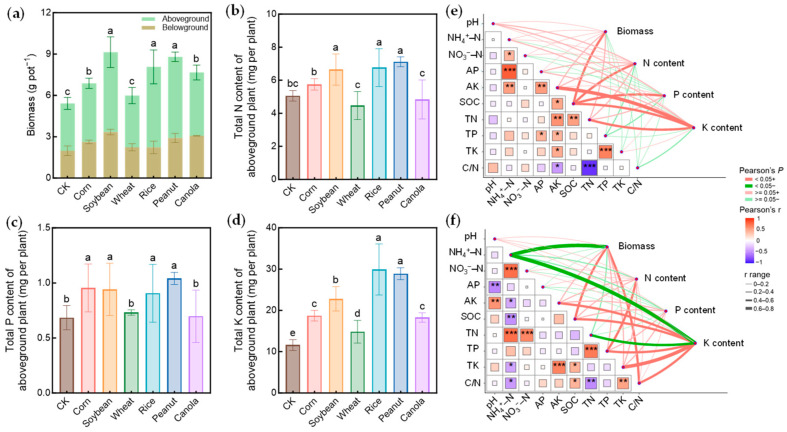
Effects of straw compost products on corn biomass (**a**), total N (**b**), P (**c**), and K (**d**) contents in aboveground tissues. Pearson correlation heatmaps between plant traits and soil physicochemical properties in bulk (**e**) and rhizosphere (**f**) soils. Values are the mean ± SD of four replicates. Different lowercase letters above bars indicate significant differences among treatments (*p* < 0.05). In the heatmaps, asterisks denote significance levels (* *p* < 0.05; ** *p* < 0.01; *** *p* < 0.001).

Compared with the control (CK), straw compost products significantly increased corn biomass (*p* < 0.05), except for the wheat compost treatment. Biomass increased by 11–69% relative to CK, following the following order: soybean (69%) > peanut (62%) > rice (49%) > canola (42%) > corn (27%) > wheat (11%) (Figure 1a). The compost products of rice, peanut, and soybean significantly increased total N content by 31–41% and total P content by 32–52% in the aboveground plants compared with CK (*p* < 0.05; Figure 1b,c). In addition, corn straw compost significantly increased total P content by 39% relative to CK (*p* < 0.05). Total K content exhibited the strongest response to compost application, increasing significantly in all treatments relative to CK (*p* < 0.05; Figure 1d), particularly in the rice and peanut treatments, with increases of 158% and 149%, respectively.

Two-way ANOVA showed that compost products and rhizosphere effects significantly influenced (*p* < 0.05 or *p* < 0.01) soil pH, NH_4_^+^–N, available P (AP), available K (AK), and soil organic carbon (SOC) (Table S1). Straw compost products also significantly (*p* < 0.05) affected the soil C/N ratio, whereas rhizosphere effects significantly influenced NO_3_^−^–N and total P (TP). The rhizosphere effect decreased soil pH, NH_4_^+^–N, NO_3_^−^–N, and AK contents (*p* < 0.05). Compared to CK, straw compost products did not affect (*p* > 0.05) soil pH and NH_4_^+^–N but increased AK content (*p* < 0.05).

Corn biomass was positively (*p* < 0.05) correlated with SOC in both bulk and rhizosphere samples and the C/N ratio in rhizosphere soil (Figure 1e,f). Corn N and P contents were positively (*p* < 0.05) correlated with SOC in bulk soil, and N content was also positively correlated with TP in rhizosphere soil (*p* < 0.05). Corn K content showed the positive correlations (*p* < 0.05) with AK and SOC in both bulk and rhizosphere soils, total N (TN) in bulk soil, and TP, total K (TK), and C/N ratio in rhizosphere soil.

### 2.3. Bacterial Community Composition and Diversity

**Figure 2:**
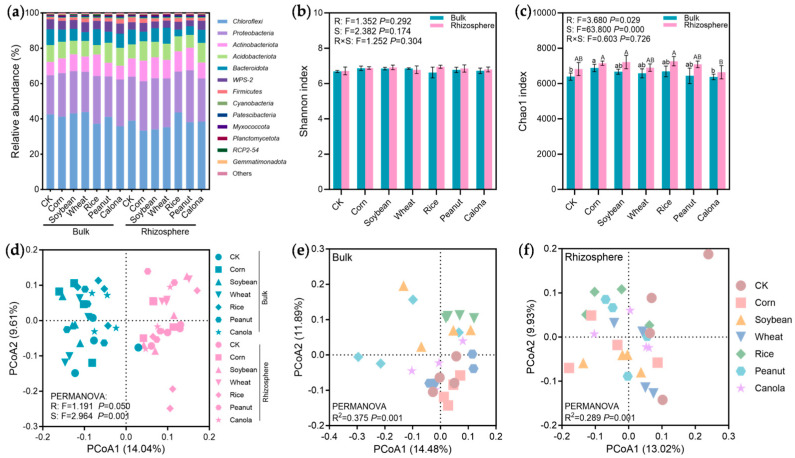
The relative abundances of the dominant bacterial phyla (≥1%) (**a**), Shannon index (**b**), Chao1 index (**c**), principal coordinate analysis (PCoA) of bacterial communities in all samples (**d**), bulk soil (**e**), and rhizosphere soil (**f**). Values are the mean ± SD of four replicates. Different lowercase and capital letters above the blue (bulk) and pink (rhizosphere) bars indicate significant differences among treatments, respectively (*p* < 0.05). The abbreviations “S” and “R” indicate straw compost products (S) and rhizosphere (R, bulk vs. rhizosphere), respectively.

At the phylum level, bacterial communities across all soil samples were dominated by *Chloroflexi* (33.30–43.83%), *Proteobacteria* (22.2–29.43%), *Actinobacteriota* (7.56–12.69%), *Acidobacteriota* (5.37–11.33%), and *Bacteroidota* (3.78–9.06%) (Figure 2a). Two-way ANOVA showed that neither compost products nor rhizosphere effects influenced the Shannon index (*p* > 0.05; Figure 2b), while they showed significant effects on the Chao1 index (*p* < 0.05; Figure 2c). Among the compost product treatments, only bulk soil amended with corn compost product exhibited a higher Chao1 index than the CK (*p* < 0.05). Across all treatments, the Chao1 index was consistently higher in rhizosphere soil compared to bulk soil, although the differences were not statistically significant.

PERMANOVA showed that rhizosphere effects (*p* = 0.001) explained a greater proportion of variation in bacterial community structure than straw compost products (*p* < 0.05). Consistently, PCoA ordination revealed a clear separation of bacterial communities between rhizosphere and bulk soils (Figure 2d). By analyzing only bulk or rhizosphere samples, straw compost products significantly altered bacterial community structure (*p* = 0.001; Figure 2e,f). The bulk bacterial communities of rice, soybean, and wheat treatments were clearly distinct from bulk sample of CK (Figure 2e), and rhizosphere bacterial communities of corn, rice, and peanut treatments showed greater separation from rhizosphere sample of CK (Figure 2f).

### 2.4. Correlation Among Bacterial Communities and Corn Growth

**Figure 3:**
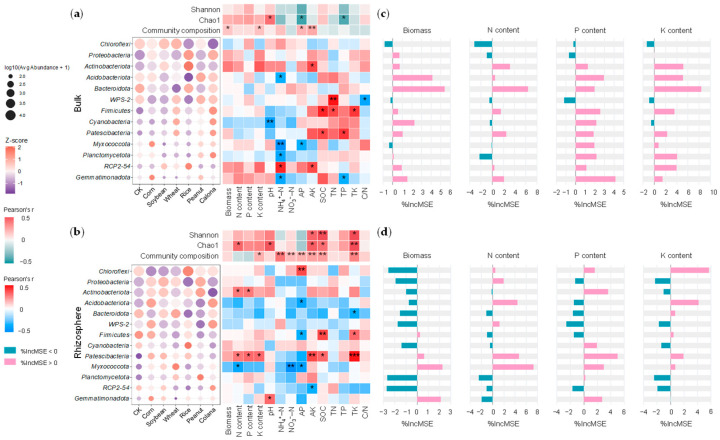
Correlations among dominant bacterial phyla, soil physicochemical factors, and corn nutrient content from bulk (**a**) and rhizosphere samples (**b**). Random forest analysis showing the contribution of each phylum to biomass and nutrient content in bulk (**c**) and rhizosphere samples (**d**). Bubble plots show average relative abundance of dominant bacterial phyla across treatments. Heatmaps represent Pearson correlations between phyla, diversity indices, corn traits, and soil physicochemical factors. Asterisks indicate significance levels (* *p* < 0.05; ** *p* < 0.01; *** *p* < 0.001).

Corn biomass and nutrient content (N, P, and K) did not show significant correlations (*p* > 0.05) with the relative abundances of dominant bacterial phyla or with α-diversity indices in bulk soil (Figure 3a). Corn biomass and K content were significantly associated (*p* < 0.05) with the bacterial community composition (Figure 3a). In the rhizosphere, corn N content was positively correlated with bacterial Chao1 index (*p* < 0.05), and K content was significantly associated with bacterial community structure (Figure 3b). The relative abundance of *Actinobacteriota* was positive correlations with corn N and P content (*p* < 0.05). *Patescibacteria* exhibited positive correlations (*p* < 0.05) with corn N, P, and K content and soil AK, SOC, and TK. The relative abundance of *Actinobacteriota* in the rhizosphere was higher in most compost treatments (corn, soybean, rice, wheat, and peanut) than in CK, whereas *Patescibacteria* was enriched across all treatments of compost products compared to CK (Figure 3b).

Random forest analysis identified *Acidobacteriota* and *Bacteroidota* as the most important predictors of corn biomass in bulk soil, whereas *Myxococcota* and *Gemmatimonadota* were the top predictors in the rhizosphere (Figure 3c,d). For corn N content, *Bacteroidota* contributed most strongly in bulk soil, while *Myxococcota*, *Patescibacteria*, and *Actinobacteriota* collectively showed the importance in the rhizosphere. Corn P content was primarily associated with *Gemmatimonadota* and *Acidobacteriota* in bulk soil, but shifted to *Patescibacteria* and *Actinobacteriota* in the rhizosphere. Regarding corn K content, *Bacteroidota* ranked first in importance in bulk soil, whereas *Chloroflexi* and *Acidobacteriota* were more influential in the rhizosphere (Figure 3c,d).

### 2.5. Microbial Co-Occurrence Networks

**Figure 4:**
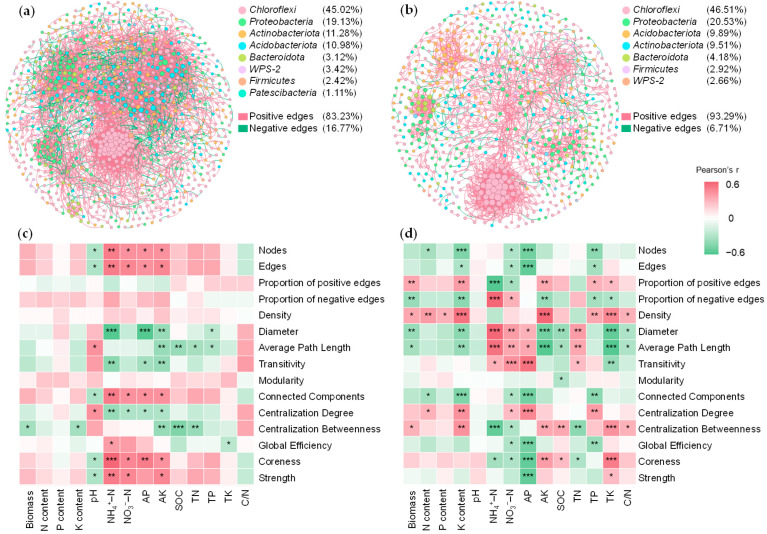
Co-occurrence networks of the bacterial communities in bulk (**a**) and rhizosphere (**b**) soils. Node colors represent phyla and sizes reflect relative abundance. Heatmaps of Pearson correlations between network topological indices and soil physicochemical factors from bulk (**c**) and rhizosphere networks (**d**). Asterisks indicate significance levels (* *p* < 0.05; ** *p* < 0.01; *** *p* < 0.001).

Microbial co-occurrence networks were constructed separately for bulk and rhizosphere samples (Figure 4a,b). Compared to the bulk network, the rhizosphere network exhibited higher diameter, average path length, transitivity, modularity, number of connected components, and betweenness centralization but lower numbers of nodes and edges, global efficiency, coreness, and node strength (Table S2). In both networks, *Chloroflexi*, *Proteobacteria*, *Actinobacteriota*, and *Acidobacteriota* accounted for the largest proportions of nodes (Figure 4a,b). Positive correlations predominated in both networks, with a higher proportion in the rhizosphere network (93.23%) than in the bulk network (83.23%).

Corn biomass and nutrient content did not show significant associations (*p* > 0.05) with topological parameters of bulk network (Figure 4c), while they were significantly and positively associated (*p* < 0.05) with four topological parameters of rhizosphere network, including the proportion of positive edges, network density, degree centralization, and betweenness centralization (Figure 4d). In the rhizosphere network, the proportion of positive edges and network density were positively correlated (*p* < 0.05) with soil AK, TP, and TK. Degree centralization was positively correlated with AP and TP, while betweenness centralization was positively correlated (*p* < 0.05) with soil AK, TK, and SOC.

### 2.6. Predicted Functional Potential of Bacterial Communities

**Figure 5:**
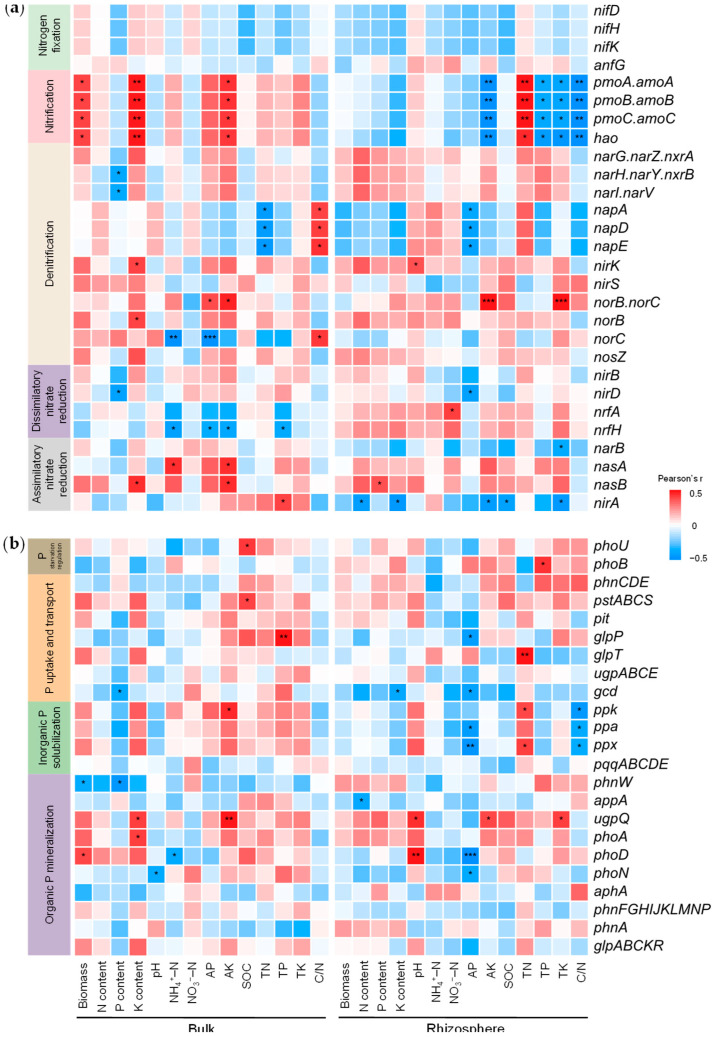
Correlation analysis between predicted functional genes involved in N (**a**) and P (**b**) cycling, corn traits, and soil physicochemical factors in bulk and rhizosphere soils. Asterisks indicate significance levels (* *p* < 0.05; ** *p* < 0.01; *** *p* < 0.001).

In bulk soil, the predicted abundances of nitrification-related genes (*pmoA/B/C*, *amoA/B/C*, and *hao*) were positively correlated (*p* < 0.05) with corn biomass, K content, and soil AK (Figure 5a). However, these genes showed negative or non-significant associations with corn biomass and nutrient content in the rhizosphere. The abundances of denitrification-related periplasmic nitrate reductase genes (*napA*, *napD*, and *napE*) in the rhizosphere were negatively associated with corn biomass and nutrient content and were also negatively correlated with soil AP (*p* < 0.05; Figure 5a). *nirA* abundance in the rhizosphere showed negative correlations (*p* < 0.05) with corn N and P content. For P cycling, corn biomass was positively correlated (*p* < 0.05) with the predicted abundance of *phoD* gene in bulk soil but negatively correlated with *phnW* gene (Figure 5b). Corn P content was negatively correlated (*p* < 0.05) with abundances of *gcd* and *phnW* genes in bulk soil but positively correlated (*p* < 0.05) with *phnW* abundance in the rhizosphere (Figure 5b).

## 3. Discussion

To elucidate the potential value of straw compost products in acidic soils, this study compared the effects of different compost straw products on soil properties and corn nutrient uptake. The results suggested that straw compost application in an acidic soil showed different effects on corn growth, which were closely associated with rhizosphere microorganisms. This study provides effective improvement measures for crop growth in acidic soils and highlights their association with rhizosphere microbial community dynamics.

### 3.1. Differential Improvement Effects of Straw Compost Products on Acidic Soil

Acidic soils are typically characterized by low pH and organic matter content, depletion of base cations, and aluminum toxicity, which severely limit crop growth and nutrient utilization [24]. Converting agricultural residues into soil amendments is a feasible strategy for achieving sustainable nutrient cycling and mitigating soil acidification. This study confirmed that applying straw compost products could improve acidic soil conditions and promote corn growth and nutrient uptake (Figure 1 and Table S1). Theoretically, the improvement effect of straw compost products on acidic soils should stem from the alkaline substance input to neutralize acidity. However, there was no significant increase in soil pH in this study (Table S1), suggesting that the ameliorative effects were not reflected in soil acidity reduction but in other pathways. The slight changes in soil pH were likely attributable to the relatively low application rate of straw compost products. Notably, the compost application rate of 5 g kg^−1^ dry soil used in this pot experiment corresponds to an equivalent field application rate of approximately 10–12 t ha^−1^, which falls within the typical agronomic range (5–20 t ha^−1^) commonly adopted for organic amendments in acidic soils. Moreover, with China’s annual straw production of nearly 900 million tons [1,2], ample crop residues are available to support such composting practices at scale. Due to the close relationship between soil pH and exchangeable Al^3+^, exchangeable Al^3+^ or base saturation were not determined [25]. Therefore, the explanation for Al detoxification remains speculative. K supply appeared to be strongly associated with corn growth in this study (Figure 1). Unlike N and P, K in crop residues is primarily present in non-structural and highly mobile forms and can be directly released into the soil solution during straw compost decomposition. In addition, compost application promoted root growth, which could expand the root–soil contact interface and improve plant nutrient uptake. K deficiency is a common limiting factor in highly weathered red soils due to intense leaching and low mineral reserves [7]. Straw compost products provide a pool of readily available base cations (K^+^, Ca^2+^, and Mg^2+^) that can rapidly alleviate the stoichiometric constraints [3]. In parallel, the accumulation of soil SOC may mitigate aluminum toxicity through the formation of stable organo–Al complexes [26]. Consistent with this interpretation, corn biomass was closely associated with both soil AK and SOC (Figure 1e). Thus, straw compost products can be regarded as a resource utilization pathway that is simultaneously associated with enhanced nutrient supply and potential alleviation of Al toxicity in acidic soils. Compared to the short-term N sequestration resulting from direct incorporation of fresh straw, straw compost products are more likely to meet the practical demands of acidic soil improvement in a relatively low-risk manner.

Although straw compost products generally benefited crop growth, significant differences were observed among straw types. Legume-derived compost products (soybean and peanut) consistently performed better than cereal straw (Figure 1). This should be attributable to inherent variations in nutrient stoichiometry and the degradability of crop straw [27]. Legume straw, characterized by a low C/N ratio and high N content, facilitates rapid mineralization and release of nutrients, thereby avoiding the risks of microbial N immobilization [7]. Cereal crops such as wheat and corn usually exhibit a high C/N ratio and low ash alkalinity [28]. Even after composting (Table 1), their compost products may also induce transient competition for N between soil microorganisms and plant roots [29]. Notably, the rice compost product performed relatively well among cereal straw. This may be attributed to its relatively low C/N ratio, and high K and silicon contents, which are known to enhance plant tolerance to stresses in acidic environments [12,30,31,32,33]. The results of this study suggested the importance of selecting straw compost products for improving acidic soils. Such an approach to straw utilization can maximize agronomic benefits while minimizing the risks of nutrient imbalance [34,35]. Overall, these differential effects likely reflect a combination of chemical (e.g., base cation content), biochemical (e.g., organic ligands mitigating Al toxicity), and biological (e.g., inoculation of functional microbes) properties inherent to each feedstock type.

### 3.2. Contribution of Rhizosphere Microorganisms to Crop Growth

The improvement effects of straw compost products on acidic soils are not limited to inputting nutrients, organic matter, and alkaline substances. The rhizosphere microorganisms should be given attention due to their role in altering the supply pattern of resources. As a microenvironment enriched with root exudates, the rhizosphere is a hotspot of microbial activity for promoting nutrient activation [12]. The microbial community in the rhizosphere is more sensitive to fertilization and amendments than bulk soil [36,37]. In this study, the rhizosphere effect exerted a stronger influence on bacterial community structure than straw compost products (Figure 2d), suggesting that the beneficial effects of compost products are likely associated with rhizosphere-specific microbial communities or metabolic processes. The rhizosphere environment affected by straw compost products could shape microbial assembly via resource filtering, favoring specific microbial taxa [11].

In the rhizosphere, *Actinobacteriota* and *Patescibacteria* emerged as core bacterial taxa associated with corn nutrient uptake, but their roles are likely distinct (Figure 3). *Actinobacteriota* showed associations more to corn N and P uptake, and similar findings have been reported [32,38]. Studies found that *Actinobacteriota* actively participated in organic matter decomposition and maintained high metabolic activity in response to organic input [30]. In this study, the enrichment of rhizosphere *Actinobacteriota* following straw compost products reflected the promoting effect of the rhizosphere on eutrophic microbial taxa [18]. In contrast, the ecological role of *Patescibacteria* relies more on a nutrient-deficiency strategy [32,39]. Members of *Patescibacteria* usually have low biosynthetic ability and require cooperation with other microbial groups to take part in organic matter composting [40].

Additionally, this study provided ecological insights into the cooperative patterns of rhizosphere microbial communities (Figure 4). Following straw compost products, the rhizosphere network exhibited a higher proportion of positive correlations and increased modularity, indicating reduced competitive pressure and enhanced metabolic complementarity among microbial species [41]. Such cooperative networks may reflect efficient connections between resources and microbial communities. Furthermore, the stable cooperative patterns of microbial communities can facilitate functional performance of nutrient-deficiency taxa such as *Patescibacteria* [42,43]. Therefore, straw compost products reshaped rhizosphere microbial communities through microbial interactions, which may be associated with nutrient transformation and retention in the root zone.

### 3.3. Microbial Processes of N and P Transformations

Based on the functional prediction of microbial communities, this study indicated a clear spatial differentiation in N and P transformations between bulk and rhizosphere soils (Figure 5). Straw compost products enhanced the predicted potential associated with nitrification in bulk soil, accelerating conversion of ammonium to nitrate. Although the active nitrification process enhances N availability, it may also raise the risk of nitrate leaching or denitrification losses [44]. In contrast, nitrification-related functional genes in the rhizosphere showed negative or insignificant correlations with crop N content. Notably, the abundance of *nirA* gene, which encodes assimilatory nitrate reductase, was reduced in response to straw compost products (Figure 5a). These changes suggest that predicted N transformation processes in the rhizosphere might been inhibited, thereby reducing the risk of N loss and benefiting plant N utilization. Such phenomenon should be attributed to nitrification inhibitors secreted by plant roots [39,45]. Evidently, the rhizosphere microenvironment prioritizes plant N uptake over microbial N cycling.

Regarding P cycling, the responses of functional genes revealed different strategies for activating P pools. In bulk soil, corn biomass correlated positively with *phoD* abundance, highlighting the role of organic P mineralization in maintaining P supply [46,47]. By contrast, corn P uptake was closely linked to *phnW* enrichment in the rhizosphere (Figure 5b). The *phnW* gene encodes enzymes responsible for cleaving recalcitrant carbon–phosphorus bonds in phosphonates, which are not readily accessible [38,48]. Thus, rhizosphere microorganisms may be involved in the inaccessible P pools. Consistent with this interpretation, previous studies have shown that straw-derived organic matter can selectively enrich phosphonate-degrading taxa, thereby expanding the pool of plant-available P [38,49]. These findings pointed to a functional differentiation between bulk and rhizosphere soils. Rhizosphere microorganisms tend to enhance the retention and activation of soil nutrients, thereby promoting crop nutrient uptake following the application of straw compost products.

## 4. Materials and Methods

### 4.1. Composting of Crop Straws

Six straw crops were selected, including corn, soybean, wheat, rice, peanut, and canola. These straws were collected from typical farmlands in Shandong Province, China. The straws were air-dried, ground, and passed through a 1 mm sieve. Straw compost products were prepared following the method described by Pan [7], in which the EM was used to accelerate the composting process. For each straw type, 500 g was mixed thoroughly with 2% (*w*/*w*) effective microorganisms (EM). Deionized water was added to adjust the moisture content to approximately 65%. The mixtures were placed in black plastic containers and subjected to aerobic composting at 28 °C under dark conditions for 45 days. During composting, the materials were manually turned every 5 days to ensure adequate aeration and homogeneous composting. After composting, the products were air-dried and used for subsequent pot experiments.

### 4.2. Pot Experiment

The acidic soil used in this study was collected from an agricultural field at the Yingtan Red Soil Ecological Experimental Station, Jiangxi Province, China (28°14′ N, 117°03′ E). The soil is developed from Quaternary red clay. After plant residues and stones were removed, the soil was air-dried, homogenized, and passed through a 2 mm sieve. Seven treatments were established, including six straw compost treatments and one control (CK) without compost application. Each compost product was uniformly mixed with 2.5 kg of dry soil at an application rate of 5 g kg^−1^ soil per pot. The mineral fertilizers were applied following conventional fertilization practices, where 0.76 g (NH_4_)_2_SO_4_ kg^−1^ soil and 0.44 g KH_2_PO_4_ kg^−1^ soil were thoroughly mixed into the soil. Each treatment consisted of eight replicates, with four planted with corn and four left unplanted. The planted and unplanted pots were used for collecting rhizosphere and bulk soils, respectively. Corn (*Zhengdan 958*) was used as the test crop. Seeds were surface-sterilized with 10% hydrogen peroxide for 10 min, rinsed three times with sterile water, and then sown. Five seeds were sown per pot. After emergence, seedlings were thinned to retain four uniform plants. Throughout the experiment, soil moisture was maintained at 60% of maximum water-holding capacity (WHC). Maximum WHC was determined gravimetrically by saturating the soil with water, allowing free drainage for 24 h, and then weighing the soil. Pots were weighed every 2 days, and deionized water was added to restore 60% WHC. All pots were placed on trays, and no visible drainage or leachate was observed during irrigation, indicating that nutrient leaching was negligible [21]. The pot experiment was conducted under natural light conditions, with temperatures ranging from 18 °C to 32 °C and relative humidity maintained between 40% and 80%.

### 4.3. Sample Collection

Plants and soils were sampled after 45 days of corn sowing. Whole plants were carefully excavated, and loosely adhering soil around the roots was gently shaken off. Soil tightly attached to the root surface was collected as rhizosphere soil. Soil collected from pots without plants was defined as bulk soil to serve as the baseline for isolating rhizosphere effects. Soil samples obtained from the same pot were homogenized as one biological replicate. All soil samples were passed through a 2 mm sieve and divided into three portions. One portion was immediately stored at −80 °C for soil DNA extraction, the second portion was stored at 4 °C for the determination of ammonium (NH_4_^+^–N) and nitrate nitrogen (NO_3_^−^–N), and the third portion was air-dried for the analysis of other soil physicochemical properties. At the same time, shoots and roots of corn were harvested separately from each pot for the determination of plant biomass and nutrient content.

### 4.4. Determination of Plant Nutrients and Soil Physicochemical Properties

Plant samples were rinsed thoroughly with deionized water, heated immediately at 105 °C for 30 min, and then oven-dried at 85 °C to constant weight. The dried samples were ground to pass through a 1 mm sieve. Shoot samples were digested using the H_2_SO_4_-H_2_O_2_ digestion method. N content was determined using the Kjeldahl method, P content was measured using the Bray method, and K content was determined using flame photometry (FP640, Shanghai, China). Soil properties including pH, SOC, TN, TP, TK, NH_4_^+^–N, NO_3_^−^–N, AP and AK were determined following the methods described by Lu [50].

### 4.5. High-Throughput Sequencing and Bioinformatic Analysis

Total soil DNA was extracted from 0.5 g of fresh soil using the FastDNA^®^ SPIN Kit for Soil (MP Biomedicals, Irvine, CA, USA) according to the manufacturer’s instructions. The V3–V4 region of the bacterial 16S rRNA gene was amplified using the primer pair 341F (5′-CCTACGGGNGGCWGCAG-3′) and 806R (5′-GGACTACHVGGGTWTCTAAT-3′). PCR amplification conditions and purification procedures followed the method described by Klindworth (2013) [51]. Purified amplicons were sequenced using paired-end sequencing on the Illumina MiSeq PE250 platform (Biozeron Biotechnology Co., Ltd., Shanghai, China).

Raw sequencing reads were quality-filtered and merged using Trimmomatic (v0.39) and FLASH (v1.2.11), respectively. Low-quality sequences (quality score < 20 or length < 50 bp) and chimeric sequences were removed using UCHIME (v8.1). The remaining high-quality sequences were clustered into operational taxonomic units (OTUs) at 97% sequence similarity using the UPARSE algorithm (v7.0.1). Taxonomic assignment of representative OTU sequences was performed using the RDP Classifier with a Bayesian algorithm against the SILVA database (v138). To minimize sampling bias caused by unequal sequencing depth, the OTU table was rarefied to 45,000 sequences per sample. The functional potential of bacterial communities was predicted using PICRUSt2 (v2.3.0) [52]. Functional genes related to N and P cycling were identified and annotated based on the MetaCyc database [53].

### 4.6. Co-Occurrence Network Analysis

Microbial co-occurrence networks were constructed for bulk and rhizosphere soils based on the OTU tables. To ensure robustness, only core OTUs detected in ≥8 samples were retained. Pairwise Spearman’s rank correlations were computed, and *p*-values were adjusted for multiple testing using the Benjamini–Hochberg false discovery rate (FDR) procedure. Only strong (|r| > 0.6) and statistically significant (FDR-adjusted *p* < 0.05) correlations were retained to construct the networks [54]. Network construction and topological analysis were performed using the igraph package in R (v4.4.3), and visualizations were generated using Gephi (v0.9.2).

### 4.7. Statistical Analysis

Statistical analyses were performed using SPSS 20.0 (SPSS Inc., Chicago, IL, USA). Effects of straw compost products on corn biomass, nutrient content, soil physicochemical properties, and microbial α-diversity indices (Shannon and Chao1) were evaluated using one-way analysis of variance (ANOVA) with the Duncan test. To assess the effects of straw compost products and soil compartments (rhizosphere vs. bulk soil), two-way ANOVA was conducted. When significant main effects or interactions were detected (*p* < 0.05), differences among treatments were further examined using the Duncan test. Microbial β-diversity was assessed based on Bray–Curtis dissimilarity matrices and visualized using principal coordinates analysis (PCoA). The effects of straw compost products and soil compartments on microbial community structure were tested using permutational multivariate analysis of variance (PERMANOVA). Mantel tests were performed to examine correlations between community composition, soil physicochemical properties, and plant traits. Pearson correlation analysis was used to explore relationships among soil properties, plant traits, relative abundances of dominant phyla, network topological parameters, and predicted functional genes. PCoA, PERMANOVA, Mantel tests, and correlation analyses were conducted in R (v4.4.3) using the vegan package. Random forest models were constructed in R using the random forest package (v4.7-1.1) [55], and variable importance was evaluated based on the percentage increase in mean squared error (%IncMSE).

## 5. Conclusions

This study demonstrates that straw compost products can serve as an effective soil amendment for enhancing corn growth and nutrient uptake in acidic soils, with efficacy varying among straw types. Straw compost addition was associated with shifts in rhizosphere microbial community composition and functional potential, and these shifts were further associated with enhanced N and P cycling and greater nutrient uptake by corn. Collectively, these findings highlight a consistent association among straw compost amendment, rhizosphere microbial community characteristics, and improved crop performance. Given the methodological constraints and the short-term experiment, the observed relationships are correlative and do not constitute direct evidence of causation. Future field studies should evaluate optimal application rates to validate these associations under realistic agricultural conditions.

## Figures and Tables

**Table 1:** Physicochemical properties of straw compost products.

Straw Type	pH	SOC (g kg^−1^)	TN (g kg^−1^)	TP (g kg^−1^)	TK (g kg^−1^)	C/N
Corn	8.29 ± 0.02	259.30 ± 19.35	17.34 ± 3.36	2.16 ± 0.08	27.63 ± 1.04	24.03 ± 6.37
Soybean	9.89 ± 0.04	261.14 ± 3.22	21.88 ± 1.31	1.51 ± 0.12	21.23 ± 1.56	19.63 ± 4.22
Wheat	7.83 ± 0.02	243.80 ± 9.55	11.92 ± 3.17	0.70 ± 0.08	25.86 ± 0.70	27.96 ± 3.73
Rice	8.91 ± 0.03	248.45 ± 14.48	26.37 ± 0.47	4.17 ± 0.15	46.34 ± 2.71	14.13 ± 0.82
Peanut	10.17 ± 0.03	243.60 ± 2.27	24.01 ± 0.84	2.49 ± 0.20	44.02 ± 1.18	15.24 ± 0.63
Canola	7.61 ± 0.03	241.20 ± 8.97	15.26 ± 0.58	0.89 ± 0.07	20.93 ± 1.69	23.76 ± 1.52

Note: Data are mean ± standard deviation (*n* = 4).

## Data Availability

The raw sequencing data generated in this study have been deposited in the NCBI Sequence Read Archive (SRA) under the BioProject accession number PRJNA1393421. Other data supporting the findings of this study are available from the corresponding author upon reasonable request.

## Supplementary Materials

The following supporting information can be downloaded at: https://www.mdpi.com/article/10.3390/plants15060879/s1, Table S1: Physicochemical properties of bulk and rhizosphere soils of maize following application of straw compost products; Table S2: Topological indices of microbial co-occurrence networks in bulk and rhizosphere samples.
