# Supplementary material for: Straw Compost Products Improve Corn Growth in Association with Rhizosphere Microbial Community in Acidic Soil
Source: Plants (Basel). 2026 Mar 12;15(6):879. doi: 10.3390/plants15060879 (PMC13030678; doi:10.3390/plants15060879)
Supplement: Supplementary file 1 [file plants-15-00879-s001.zip › plants-4116665-supplementary.pdf]

Table S1 Physicochemical properties of bulk and rhizosphere soils of maize following application of straw compost products

Properties	Sampling Site	CK	Corn	Soybean	Wheat	Rice	Peanut	Canola	Two-way ANOVA
pH	Bulk	**4.55 ± 0.28ab**	**4.61 ± 0.05a**	**4.45 ± 0.01ab**	**4.42 ± 0.05ab**	**4.53 ± 0.04b**	**4.54 ± 0.05ab**	**4.37 ± 0.01ab**	S: F=2.46* , R: F=80.41**,
	Rhizosphere	**4.22 ± 0.04b**	**4.29 ± 0.06ab**	**4.33 ± 0.07a**	**4.23 ± 0.05b**	**4.23 ± 0.05ab**	**4.29 ± 0.06ab**	**4.21 ± 0.03b**	S × R: F=1.34**
NH_4^+-N (mg kg^{-1})	Bulk	**11.59 ± 2.81ab**	**1.34 ± 0.81d**	**11.07 ± 1.20abc**	**10.26 ± 0.64bc**	**14.43 ± 2.48a**	**10.23 ± 2.81bc**	**7.79 ± 0.84c**	S: F=15.25** , R: F=234.02**,
	Rhizosphere	**5.94 ± 0.44a**	**3.46 ± 0.22b**	**1.78 ± 0.05d**	**2.90 ± 0.02bc**	**2.97 ± 0.76bc**	**1.99 ± 0.66d**	**2.11 ± 0.54cd**	S × R: F=14.71**
NO_3^--N (mg kg^{-1})	Bulk	**1.23 ± 0.15ab**	0.82 ± 0.43b	**1.45 ± 0.32a**	**1.19 ± 0.11ab**	0.93 ± 0.14ab	1.21 ± 0.35ab	1.03 ± 0.37ab	S: F=1.80 , R: F=100.56**,
	Rhizosphere	**0.72 ± 0.02a**	0.49 ± 0.09b	**0.29 ± 0.03c**	**0.28 ± 0.02c**	0.62 ± 0.02ab	0.51 ± 0.21b	0.27 ± 0.08c	S × R: F=3.12*
AP (mg kg^{-1})	Bulk	15.68 ± 0.50b	**4.83 ± 0.56c**	12.51 ± 1.44b	13.92 ± 0.37ab	19.85 ± 2.69a	14.19 ± 2.13ab	**12.82 ± 0.73ab**	S: F=30.74** , R: F=108.12**,
	Rhizosphere	19.62 ± 1.78a	**15.39 ± 1.00c**	15.29 ± 0.71c	16.37 ± 1.26bc	18.31 ± 1.22ab	19.52 ± 1.40a	**18.22 ± 1.46ab**	S × R: F=12.26**
AK (mg kg^{-1})	Bulk	**18.52 ± 0.55c**	**17.80 ± 1.68c**	**28.59 ± 4.00b**	**29.28 ± 1.07b**	**38.01 ± 3.09a**	**35.42 ± 2.82a**	**27.56 ± 4.04b**	S: F=37.49** , R: F=561.17**,
	Rhizosphere	**4.99 ± 0.99d**	**13.01 ± 0.70b**	**9.67 ± 0.43c**	**14.42 ± 0.89ab**	**16.61 ± 2.41a**	**15.58 ± 3.54ab**	**8.61 ± 0.95c**	S × R: F=10.075**
SOC (g kg^{-1})	Bulk	5.60 ± 0.25b	6.93 ± 0.45b	7.01 ± 0.48b	6.74 ± 0.26b	6.88 ± 0.41c	7.86 ± 0.21a	7.01 ± 0.47b	S: F=6.05** , R: F=25.49**,
	Rhizosphere	6.66 ± 0.91b	7.46 ± 0.26ab	8.43 ± 0.48a	7.49 ± 0.80ab	8.39 ± 0.64a	7.72 ± 0.44ab	7.83 ± 0.56a	S × R: F=1.76
TN (g kg^{-1})	Bulk	1.41 ± 0.41b	1.67 ± 0.41ab	2.05 ± 0.29a	1.84 ± 0.21ab	1.99 ± 0.22ab	2.29 ± 0.39a	1.95 ± 0.28ab	S: F=1.49 , R: F=0.60,
	Rhizosphere	2.63 ± 0.68a	1.67 ± 0.41ab	1.78 ± 0.49ab	1.51 ± 0.71b	1.10 ± 0.54b	1.99 ± 0.22ab	1.59 ± 0.10b	S × R: F=4.13**
TP (g kg^{-1})	Bulk	3.05 ± 0.22	2.54 ± 0.24	2.85 ± 0.32	3.38 ± 0.34	3.03 ± 0.49	3.32 ± 0.33	3.26 ± 0.32	S: F=3.93** , R: F=15.06**,
	Rhizosphere	3.21 ± 0.23	3.17 ± 0.46	3.31 ± 0.25	1.48 ± 1.90	1.60 ± 1.97	3.74 ± 0.10	3.37 ± 0.19	S × R: F=1.13
TK (g kg^{-1})	Bulk	107.48 ± 2.78ab	106.77 ± 5.84ab	103.69 ± 8.18ab	113.78 ± 5.10ab	114.10 ± 5.87ab	115.77 ± 9.48ab	113.54 ± 1.58ab	S: F=3.66** , R: F=1.53,
	Rhizosphere	100.71 ± 4.15d	109.34 ± 2.11bc	112.24 ± 0.84ab	56.06 ± 53.26bc	59.18 ± 56.34a	109.45 ± 1.46bc	106.47 ± 2.01c	S × R: F=2.65*
C/N	Bulk	8.48±2.36	8.47±3.23	6.49±0.94	6.84±0.63	6.46±0.62	6.53±0.89	6.80±1.05	S: F=1.47 , R: F=9.56**,
	Rhizosphere	4.92±1.05b	9.55±4.45ab	9.93±4.28ab	10.92±3.89ab	17.85±7.99a	9.99±4.62ab	9.43±0.21ab	S × R: F=2.59*

Note: Values are means ± standard deviation (n=4). Different letters in the same row indicate significant differences (Duncan's test; P<0.05). Bold font indicates

significant differences between bulk and rhizosphere samples within the same treatment ($P<0.05$). (*$P < 0.05$; **$P < 0.01$). S: Application of composted straw; R: Rhizosphe re effect.

Table S2 Topological indices of microbial co-occurrence networks in bulk and rhizosphere samples

	Bulk	Rhizosphere
Nodes	993	789
Edges	4849	2222
Proportion of positive edges	83.23%	93.29%
Proportion of negative edges	16.77%	6.71%
Density	0.01	0.01
Diameter	8.54	10.30
Average Path Length	3.16	4.39
Transitivity	0.47	0.48
Modularity	0.66	0.73
Connected Components	18	58
Centralization Degree	0.06	0.06
Centralization Betweenness	0.03	0.09
Global Efficiency	0.22	0.13
Coreness	5.77	3.44
Strength	6.82	4.06
